# International Consensus on Reporting Anastomotic Leaks After Colorectal Cancer Surgery: The CoReAL Reporting Framework

**DOI:** 10.1097/DCR.0000000000003790

**Published:** 2025-05-07

**Authors:** Danique J.I. Heuvelings, Nicole D. Bouvy, Nader K. Francis, Sander M.J. van Kuijk, Merel L. Kimman, Marylise Boutros, Patricia Sylla, On behalf of the CoReAL Collaborative

**Affiliations:** 1 NUTRIM School of Nutrition and Translational Research in Metabolism, Maastricht University, Maastricht, The Netherlands; 2 Department of Surgery, Maastricht University Medical Center, Maastricht, The Netherlands; 3 GROW School for Oncology and Developmental Biology, Maastricht University, Maastricht, The Netherlands; 4 Division of Surgery and Interventional Science, University College, London, United Kingdom; 5 The Griffin Institute, Northwick Park and St Mark’s Hospital, Harrow, United Kingdom; 6 Department of Clinical Epidemiology and Medical Technology Assessment (KEMTA), Maastricht University Medical Centre, Maastricht, The Netherlands; 7 Department of Colon and Rectal Surgery, Cleveland Clinic Florida, Weston, Florida; 8 Division of Colon and Rectal Surgery, Icahn School of Medicine at Mount Sinai, New York, New York

**Keywords:** Anastomotic leakage, Colorectal surgery, Consensus, Patient outcomes, Reporting

## Abstract

**BACKGROUND:**

Anastomotic leak frequently complicates colorectal anastomoses with high morbidity and mortality. The substantial variability in published leak rates reflects the lack of consistency in reporting variables that may impact the occurrence, management, and short- and long-term outcomes of patients.

**OBJECTIVE:**

The Consensus for Reporting of Colorectal Anastomotic Leaks is an international collaborative that developed a standardized evidence-based framework for reporting key variables related to the entire episode of colorectal anastomotic leak in patients with cancer.

**DESIGN:**

Along the preoperative, intraoperative, and short- and long-term postoperative phases of a left-sided colorectal anastomotic leak, key questions regarding all potentially relevant variables were formulated. A literature review was conducted to generate evidence-based statements in response to these questions. Statements that reached consensus, together with input from patients’ experience and experts’ opinion, were incorporated into the framework as reporting elements.

**SETTING:**

Modified Delphi methodology, including online voting and an in-person consensus meeting, was used to generate consensus statements based on the literature review and to develop the reporting framework.

**PARTICIPANTS:**

An international panel of 32 colorectal surgeons with expertise in the field of colorectal anastomotic leaks, representing 6 surgical societies, along with radiologists, research collaborators, patients, health care economists, and surgical trial methodologists.

**MAIN OUTCOME MEASURES:**

Evidence-based statements and reporting elements with more than 70% agreement were included.

**RESULTS:**

Consensus among experts was achieved on 33 evidence-based statements and 43 reporting elements for the Consensus on Reporting colorectal Anastomotic Leaks framework. The reporting elements encompassed evidence-based statements (27), patient perspectives (7), and expert opinion (9).

**LIMITATIONS:**

Sampling did not represent all regions in the world. Because of the paucity of evidence for some topics, evidence-based statements were primarily based on a moderate-to-low level of evidence.

**CONCLUSIONS:**

This international consensus provides an evidence-based standardized framework for reporting of key variables related to a colorectal anastomotic leak after oncologic resection. See **Video Abstract**.

**CONSENSO INTERNACIONAL SOBRE LA NOTIFICACIÓN DE FUGAS ANASTOMÓTICAS TRAS LA CIRUGÍA DE CÁNCER COLORRECTAL: EL MARCO DE NOTIFICACIÓN CoReAL:**

**ANTECEDENTES:**

La fuga anastomótica complica frecuentemente las anastomosis colorrectales, con alta morbilidad y mortalidad. La considerable variabilidad en las tasas de fugas publicadas refleja la falta de consistencia en el reporte de variables que pueden afectar en los pacientes, tanto la incidencia y el manejo como los resultados a corto y largo plazo.

**OBJETIVO:**

El Consenso para el Reporte de Fugas Anastomóticas Colorrectales (CoReAL) es el resultado de una colaboración internacional que desarrolló un marco estandarizado basado en la evidencia para evaluar las variables clave relacionadas con el episodio de fuga anastomótica colorrectal en pacientes con cáncer.

**DISEÑO:**

Durante las fases preoperatoria, intraoperatoria y postoperatoria a corto y largo plazo en casos de fuga anastomótica colorrectal izquierda, se formularon preguntas clave sobre todas las variables potencialmente relevantes. Se realizó una revisión bibliográfica para generar declaraciones basadas en la evidencia como respuesta a estas preguntas. Las declaraciones consensuadas, junto con la experiencia de los pacientes y la opinión de expertos, se incorporaron al marco como elementos de reporte.

**CONTEXTO:**

Se utilizó la metodología Delphi modificada, que incluyó votación en línea y una reunión de consenso presencial, para generar declaraciones de consenso basadas en la revisión bibliográfica y desarrollar el marco de presentación de informes.

**PARTICIPANTES:**

Panel internacional que incluyó 32 cirujanos colorrectales con experiencia en el campo de las fugas anastomóticas colorrectales, cirujanos representantes de 6 sociedades quirúrgicas, junto con radiólogos, colaboradores de investigación, pacientes, economistas de la salud y metodólogos de estudios quirúrgicos.

**PRINCIPALES MEDIDAS DE RESULTADOS:**

Se incluyeron las declaraciones basadas en la evidencia y los elementos de informes con más del 70% de acuerdo.

**RESULTADOS:**

Se logró consenso entre los expertos sobre 33 declaraciones basadas en la evidencia y 43 elementos de presentación de informes para el marco CoReAL. Los elementos con la presentación de informes abarcaron declaraciones basadas en la evidencia (27), perspectivas de los pacientes (7) y la opinión de expertos (9).

**LIMITACIONES:**

El muestreo no representó a todas las regiones del mundo. Debido a la escasez de evidencia en algunos temas, las declaraciones basadas en la evidencia se basaron principalmente en un nivel de evidencia moderado a bajo.

**CONCLUSIONES:**

El presente consenso internacional proporciona un marco estandarizado basado en la evidencia para informar las variables clave relacionadas con la fuga anastomótica colorrectal después de una resección oncológica. *(Traducción—Dr. Xavier Delgadillo*)

Anastomotic leak (AL) represents a critical and challenging complication of colorectal cancer (CRC) resections with substantial impact on short- and long-term outcomes. Despite advances in preoperative risk assessment, surgical technique, and postoperative care, the incidence of colorectal AL ranges from 1.5% to 23% with sequelae that range from minimal to severe morbidity, and mortality rates as high as 16% to 29%.^1–5^ The lack of consensus on how AL is defined, graded, and reported complicates our understanding of the true incidence of AL and our ability to compare risk factors, interventions, and outcomes of AL across studies.^6,7^

In recent years, there has been a growing recognition of the importance of standardizing the reporting of colorectal ALs. A recent systematic review of the literature to assess the quality of reporting of AL in CRC trials highlighted significant heterogeneity across trials. Among studies where colorectal AL after CRC resection was a primary or secondary end point, only 20% provided clear reporting of how AL was defined.^8^ Despite the lack of a widely adopted definition, even fewer studies describe important elements for their AL cases, such as diagnostic modalities and/or reinterventions, and outcomes for AL in the short- and long-term study follow-up.^8^ This lack of reporting critical information on AL undermines the validity of clinical trials and complicates the comparison of any given interventions on outcomes of AL across studies, ultimately hindering the assessment of the effectiveness of strategies to mitigate AL.^9–12^ In addition, a prospective measure and reporting of essential pre-, intra-, and early postoperative elements can prevent or aid early detection of AL.

Although previous endeavors have been undertaken to achieve consensus on definitions and severity grading of colorectal AL, widespread adoption and reporting have remained limited.^11,13–15^ The Consensus on Reporting colorectal Anastomotic Leaks (CoReAL) project sought to improve how leaks are documented and reported by capturing contributing factors, interventions, and long-term sequelae. We hypothesized that the wide adoption of a standardized and evidence-based framework may address the systematic underreporting of colorectal AL and enhance the consistency and quality of AL reporting for both clinical practice and research.

The aim of the CoReAL project was to create a framework to standardize the reporting of AL after left-sided CRC resections with a colorectal anastomosis based on expert consensus as informed by high-level published evidence and patient perspectives.

## MATERIALS AND METHODS

**FIGURE 1.**
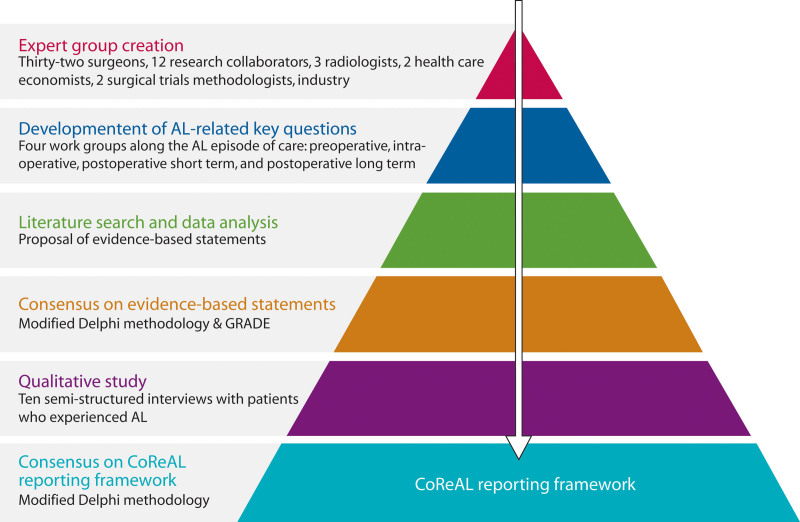
Work process and methodology of the CoReAL framework. AL = anastomotic leak; CoReAL = Consensus on Reporting colorectal Anastomotic Leaks project; GRADE = Grading of Recommendations Assessment, Development and Evaluation.

The CoReAL project consisted of 2 phases (Fig. 1). First, all available evidence regarding key questions related to factors that may or may not contribute to the development, severity, and short- and long-term outcomes of AL were analyzed and used to develop evidence-based statements. Second, the evidence statements were complemented with expert opinions and patients’ perspectives to develop a reporting framework. The topic of colorectal AL was divided into 4 phases along the AL episode of care, including preoperative, intraoperative, postoperative short-term phases, and postoperative long-term phases. A working group (WG) was created for each phase.

### Research Team

An expert panel of surgeons functioned as the team leads (P.S., N.K.F., M.B., and N.D.B.) together with a surgical research fellow (D.J.I.H.). The coordinating team extended invitations to a diverse group of colorectal expert surgeons to join the expert panel. Criteria for invitation included previously leading or contributing to surgical trials in CRC, research on colorectal AL, or participating in the development of AL guidelines. This research group comprised 32 expert surgeons and 12 research collaborators representing 6 international surgical societies; the American Society of Colon and Rectal Surgeons, the Society of American Gastrointestinal and Endoscopic Surgeons, the European Association for Endoscopic Surgery, the European Society of Coloproctology, the Endoscopic and Laparoscopic Surgeons of Asia, and the Colorectal Surgical Society of Australia and New Zealand. The research team was distributed across the 4 WGs, with balanced representation from the various surgical societies (see Appendix 1 at https://links.lww.com/DCR/C516). Experts from related specialties were consulted during the course of the project, including 3 radiologists, 4 industry representatives, and 10 patient advocates who developed an AL after colorectal resection. M.L.K. and S.M.J.v.K. served as health outcomes and trial methodologists and provided guidance throughout all phases of the project.

### Research Questions and Search Strategy

Along the 4 phases of the AL episode of care, the coordinating team developed a comprehensive list of key questions related to AL (see Supplemental Table 1 at https://links.lww.com/DCR/C518). After expert input, the final list was divided among the corresponding WGs for further investigation. A literature search was conducted to assess the evidence related to each question, led by D.J.I.H. and a librarian. The search was performed in PubMed, Embase, and Cochrane electronic libraries on November 3, 2022 (see Supplemental Table 2 at https://links.lww.com/DCR/C518). Only high-level evidence articles were selected, including randomized controlled trials and systematic reviews with or without meta-analyses in which AL after CRC surgery was a primary or secondary outcome. Eligible articles were required to be published in English after 2000. Articles that did not report on oncological left-sided colorectal resections were excluded. All search results were imported into Rayyan to allocate manuscripts to a given topic and WG, with initial eligibility determined on the basis of title and abstract review.^16^ D.J.I.H. and research collaborators performed the screening for each question. In cases of disagreement, the team leads acted as referees. Eligible full-text articles were reviewed and summarized. The search was updated on July 26, 2023.

### CoReAL Definitions

As previously demonstrated in our recent systematic review on the quality of AL reporting in CRC trials, significant heterogeneity exists in AL reporting and definitions, making any comparison between studies flawed.^8^ To overcome this limitation, the team agreed to define AL in the broadest way possible rather than to follow any specific criteria, including the International Study Group of Rectal Cancer definition. Thus, for this consensus, AL was defined as any breach or failure in the integrity of the anastomosis, including dehiscence, insufficiency, failure, breakdown, defect, or separation, regardless of the diagnostic modality (radiologic, endoscopic, intraoperative) and irrespective of clinical or biochemical manifestations. Defining the timing of AL diagnosis was considered important due to its different implications on health care resource utilization and outcomes. Based on consensus, AL was considered “early” when diagnosed ≤90 days from the index surgery and “late” or “delayed” when diagnosed after 90 days.

### Data Extraction and Evidence-Based Statements

Data extraction was conducted using RevMan Web (Review Manager Web, Computer program, version 4.12.0. The Cochrane Collaboration, 2022). General information regarding oncologic colorectal AL, including definitions, severity assessment, diagnostic time frame, clinical symptoms, biochemical tests, imaging modalities, type of reinterventions, and long-term outcomes, was collected using standardized forms. Key outcome measures related to AL (eg, relative risk, OR, HR) were extracted for every research question. If systematic reviews showed overlapping data, the lowest quality study was excluded, or a new overview was created, which only included mutually exclusive studies. If no overlap was found, data were pooled using RevMan Web tools. Methodological quality and risk of bias for included studies were assessed by 2 research collaborators using the RoB2 tool for randomized controlled trials and the ROBIS tool for systematic reviews and meta-analyses.^17,18^ Bias was visualized with a risk-of-bias visualization tool in RevMan Web.

After summarizing and presenting all the evidence related to each question, evidence-based statements were formulated by each WG to address all AL-related questions. The formulation of the statements was based on the level of evidence (LoE) according to the Grading of Recommendations Assessment, Development and Evaluation guidelines, rated as “high,” “moderate,” “low,” or “very low.”^19^ Standardized wording used to phrase statements is shown in Table 1. When the LoE was very low, the research team did not formulate a statement but flagged the topic as lacking evidence.

**TABLE 1.** Phrasing of the statements based on the LoE

*LoE according to GRADE*	*Wording statement*
High	Does (not)
Moderate	Probably does (not)
Low	May (not) do
Very low/expert opinion	No statement formulated

GRADE = Grading of Recommendations Assessment, Development and Evaluation; LoE = level of evidence.

### Consensus on Drafted Statements

A 2-phase modified Delphi method, consisting of an online survey and an in-person consensus meeting, was used to achieve consensus on all evidence-based statements. In the first phase, the statements from each WG with LoE were presented to all 32 team experts who subsequently voted online on each of these statements using a 9-point Likert scale, with consensus defined as more than 70% agreement (a score of 7 or more on the Likert scale). In the second phase, statements that did not reach consensus were discussed on day 1 of a 2-day in-person consensus meeting held in Boston in October 2023. Research collaborators presented the data analysis for all statements that did not reach consensus and facilitated discussions and rephrasing of the statements among experts. Another round of voting was performed, with consensus achieved with more than 70% agreement.

### Patient Engagement

Ten CoReAL patient partners, who experienced an AL after colorectal surgery, provided input regarding the project through in-depth semi-structured interviews. The detailed outcomes of these interviews are described in a separate qualitative article that is currently under review at *Diseases of the Colon & Rectum*.^20^ The results were presented during the in-person consensus meeting and incorporated into the reporting framework.

### Development of the Reporting Framework and Consensus Process

The reporting framework encompassing reporting elements along all 4 phases of the AL episode was constructed from the statements that achieved consensus, with input derived from qualitative analysis of patients’ interviews and from experts. The framework was developed using a modified Delphi method conducted in 2 phases. On day 2 of the in-person consensus meeting, each WG formulated a list of reporting elements derived from the consensus statements with additional elements derived from patients and experts. The reporting elements were then presented to the wider group for discussion, followed by voting. The results were consolidated to create an initial draft of the reporting framework. In the second phase, all experts were asked to rate their agreement with the inclusion of each element in the final reporting framework in an online survey using a 9-point Likert scale. Consensus was achieved with more than 70% agreement (a score of 7 or more on the Likert scale). All the reporting elements that achieved consensus were incorporated into the final CoReAL framework.

## RESULTS

The literature search yielded 2989 abstracts, of which 453 were included and analyzed. The search was updated on July 26, 2023, and yielded an additional 24 articles, for a total of 477 included articles.

### Evidence-Based Statements

The first online Delphi round was completed by 30 experts and the second round by 26 experts who attended the in-consensus meeting (23 participated in person and 3 connected remotely). By the end of day 1, 33 evidence-based statements reached consensus (Table 2). For 13 topics, the evidence was insufficient to formulate a statement (Table 3). Experts’ commentary on the consensus statements can be found in Supplemental Table 3 at https://links.lww.com/DCR/C518.

**TABLE 2.**
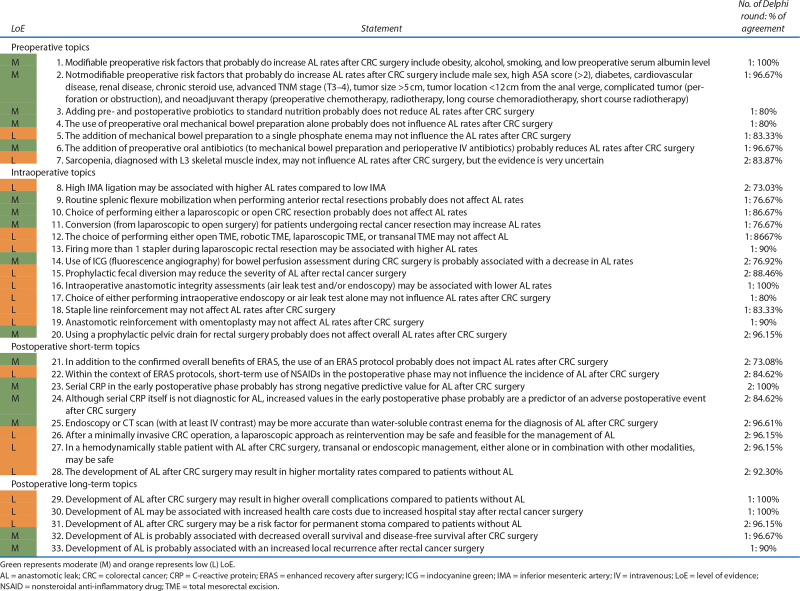
Overview of evidence-based statements with corresponding LoE and percentage of agreement

**TABLE 3.** Overview of topics that require further investigation

*Topic*	*Reason to not formulate a statement*
Preoperative	
Preoperative selective decontamination compared to broad-spectrum antibiotics	Too low level of evidence
Anemia correction	Too low level of evidence
Oral nutritional supplement/support	Too much heterogeneity in the duration of administration, different types of oral nutritional support, and no clear consensus on what is the definition of immunonutrition
Intraoperative	
Human factors^a^	Too low level of evidence
Anastomotic configuration^b^	The reported data in the analysis are very scarce and heterogeneous; overall evidence was too low
Anesthesia factors or intraoperative risk scoring systems	Too low level of evidence and lack of worldwide validation
Postoperative short term	
Clinical predictions scores	Not described in high-level evidence literature
Peritoneal fluid markers	Not described in high-level evidence literature
Low fiber diet	Not described in high-level evidence literature
Laxatives	Not described in high-level evidence literature
Postoperative long term	
Impact on QoL	Too low level of evidence
Financial impact	Too low level of evidence; the expert team believed that additional intervention, imaging modalities, and paramedical care were contributors within the statement
Chronic sequalae of AL	Evidence too low, not well described in high-level evidence literature

AL = anastomotic leak; QoL = quality of life.

a Hospital volume, surgeon volume, and surgeon specialization.

b Side-to-side vs side-to-end vs end-to-end vs J-pouch, antiperistaltic vs isoperistaltic, intracorporeal vs extracorporeal, handsewn vs stapled, immediate vs delayed (Turnbull-Cutait), compression vs handsewn vs stapled, and single- vs double-layered anastomosis.

### CoReAL Reporting Framework

By the end of day 2 of the in-person consensus meeting, 46 reporting elements were included in the reporting framework, including 7 preoperative, 14 intraoperative, 7 postoperative index admission, 8 postoperative 30 to 90 days, and 10 postoperative long-term elements. After the second online Delphi round, 3 elements did not reach consensus. Of the 43 reporting elements that reached consensus, 27 reporting elements were derived from evidence-based consensus statements, 7 were based on patient perspectives, and 9 were from expert opinion (Table 4). Patient-centered elements were informed by the results of our qualitative analysis and included preoperative discussion regarding the potential need for a stoma after surgery, preparation and planning for possible stoma creation, postoperative assessment, management of potential sequelae of AL, and the impact of AL on quality of life and functional outcomes.^21^ Consensus was reached to report these outcomes for at least 1 year after surgery, with the aspirational goal of reporting on oncologic, survival, and functional status at 2 and 5 years postoperatively. Although the evidence suggested that mechanical bowel preparation alone and routine splenic flexure mobilization did not significantly impact AL rates, both were included as elements based on expert opinion that they reflected standard practice during left-sided restorative proctectomy for cancer. In addition, 6 intraoperative elements reflecting the intraoperative difficulty (n = 3) and surgical pitfalls (n = 3) were based on expert opinion. Although these elements are not based on evidence, they were believed to serve as surrogates for the technical and human factors that likely contribute to AL. Finally, given the importance of documenting the resolution (or lack thereof) of AL and its potential sequelae, the status of the anastomosis was included as a postoperative element to be captured beyond 90 days and up to 1 year postoperatively. A detailed version of the reporting framework can be found in Appendix 2 at https://links.lww.com/DCR/C517.

**TABLE 4.** Reporting elements included in the CoReAL reporting framework

*Reporting elements*	*Agreement, %*	*Background*
Preoperative elements		
1. Modifiable risk factors	92.31	Evidence based
2. Preoperative oral antibiotics	92.31	Evidence based
3. Mechanical bowel preparation	88.45	Expert opinion
4. Other risk factors	84.62	Evidence based
5. Was the potential need of a postoperative/permanent stoma discussed?	84.62	Patient centered
6. Was the patient referred to a stoma therapist preoperatively?	76.92	Patient centered
Intraoperative elements		
7. Diverting stoma creation	100	Evidence based
8. Intraoperative difficulty: distance of the anastomosis (cm) from AV	100	Expert opinion
9. Anastomotic integrity testing	96.15	Evidence based
10. Number of stapler loads for rectal transection	92.31	Evidence based
11. Intraoperative difficulty: redo pelvic surgery	92.31	Expert opinion
12. Conversion MIS to open	88.46	Evidence based
13. Pitfalls: pelvic stapler failures	88.46	Expert opinion
14. Pitfalls: unplanned multivisceral resection or repair (of organ injury)	84.62	Expert opinion
15. Splenic flexure mobilization	84.62	Expert opinion
16. Intraoperative difficulty: acute blood loss requiring blood transfusion	80.77	Expert opinion
17. Location of inferior mesenteric artery ligation	76.92	Evidence based
18. Pitfalls: other device failures	76.92	Expert opinion
19. Perfusion assessment of conduit with fluorescence angiography	73.08	Evidence based
Reporting elements before discharge (index admission)		
20. Mortality	100	Evidence based
21. Reinterventions until discharge	100	Evidence based
22. Stoma creation	100	Evidence based
23. Diagnostic modality for AL	96.15	Evidence based
24. Length of hospital stay	92.31	Evidence based
25. Length of stay in the ICU	84.62	Evidence based
26. Serial CRP measurement	84.62	Evidence based
Reporting elements after discharge—30 d and up to 90 d		
27. Mortality	100	Evidence based
28. Readmission	100	Evidence based
29. Reinterventions after initial discharge	100	Evidence based
30. Stoma creation and closure	100	Evidence based
31. Diagnostic modality for AL	96.15	Evidence based
32. Anastomotic complication	96.15	Patient centered
33. Length of hospital stay	88.46	Evidence based
34. Length of stay in the ICU	84.61	Evidence based
Reporting elements after 90 d (long term)		
35. Reinterventions after 90 d^a^	100	Evidence based
36. Stoma information^a^	96.15	Evidence based
37. Anastomotic complications^a^	96.15	Patient centered
38.Oncological outcomes^b^	96.15	Evidence based
39. Mortality^b^	96.15	Evidence based
40. Anastomotic status^a^	92.31	Expert opinion
41. Functional outcomes: LARS (LARS score)^b^	88.46	Patient centered
42. Quality-of-life assessment (EQ-5D score)^b^	84.62	Patient centered
43. Functional outcomes: Incontinence (Wexner FI score)^b^	80.76	Patient centered

AV = anal verge; CoReAL = Consensus on Reporting Colorectal Anastomotic Leaks; CRP = C-reactive protein; EQ-5D = EuroQol 5 Dimensions; FI = fecal incontinence; ICU = intensive care unit; LARS = low anterior resection syndrome; MIS = minimally invasive surgery.

a Up to 1 y.

b At 1, 2 (when possible), and 5 y (when possible).

## DISCUSSION

Prior attempts to achieve consensus on definitions of colorectal AL have had limited success. As demonstrated in a recent systematic review of the quality of reporting of AL across CRC trials, substantial variability in the reporting of contributing factors, diagnostic modalities, interventions, and impact of colorectal AL on functional and oncologic outcomes persists. These variables are of important value not only to patients but also to administrators, quality officers, payers, and industries.^8,21–24^

The CoReAL project aimed to bridge this gap by developing a standardized reporting framework for patients undergoing left-sided CRC resections. Drawing on the most robust evidence available, along with insights from patients and experts, the CoReAL framework encompasses key variables related to the development, severity, and postoperative outcomes of AL to create a comprehensive, data-driven, and patient-centered approach for the clinical reporting of AL. We believe that integration of this framework into the clinical workflow will promote risk stratification for AL, adopt evidence-based preventive and mitigation strategies, and demonstrate that time to diagnosis and corrective intervention correlates with persistence of long-term sequelae and patient-reported outcomes.

The strength of the article derives from the rigorous methodology for consensus development among a large group of international experts with a wide range of practices. Representation of several surgical societies was critical for endorsement, dissemination, and subsequent adoption by members. The core of the project’s achievement lies in its evidence synthesis, which is encapsulated in 33 evidence-based statements derived from the highest LoE. The framework differs from prior consensus efforts in that it is largely built on these evidence-based statements and enriched by patients’ experiences and experts’ opinions to ensure it achieves its objectives while remaining relevant and meaningful to all relevant stakeholders.^13,14^

The CoReAL reporting framework consists of 43 elements, organized along the 4 phases of the AL episode. This structured approach was intended to standardize reporting practices rather than replace existing AL classification systems. We envisage the framework to become integrated into the clinical workflow, ensuring that implementation does not disrupt but complements current documentation. Preoperative elements can be included in standard assessments or informed consent discussions. Intraoperative elements can be added to operative report templates for CRC resections, whereas discharge summaries should incorporate short-term postoperative elements from the index admission and any readmissions, with follow-up reports including data up to 30 days, 90 days, and beyond.

To date, institutions have only been required to report the 30-day leak, reintervention, reoperation, and readmission rates, which have been used as colectomy-specific quality benchmarking. This requirement has reinforced the stigma associated with the reporting of AL and deterred clinical teams from interrogating anastomoses early, particularly when subclinical leaks are suspected. Another shortcoming of traditional quality reporting is that it does not consider whether steps were taken to mitigate the risk of leaks and to identify and manage them early, effectively shortening the time to resolution. Extending the reporting period beyond 90 days is also critical to document resolution of AL, assess the true impact on health care resource utilization, and capture oncologic and functional sequelae, which are often omitted in shorter follow-up periods.^25,26^ The proposed extended reporting time frame for AL, which reached consensus among experts, was aligned with patients’ feedback regarding the need for better support for patients affected by long-term sequelae of AL.

The widespread adoption of the CoReAL framework holds the potential to standardize and destigmatize the reporting of AL. By providing a clear and consistent methodology, the framework can enhance the quality of AL-related research and clinical care. This standardization will help ensure that data collected across different studies and clinical settings are comparable and reliable, facilitating better comparison of trials and meta-analyses. In addition, standardized reporting can help demystify AL for patients, providing them with clearer information regarding their prognosis and the long-term impact of their condition. The framework could also serve as a model for clinical trials using AL as a clinical end point and for benchmarking surgical outcomes and postoperative complications at the institutional level.

It is important to highlight that the CoReAL framework is not intended to replace prior AL guidelines but provides a high-quality, evidence-based, expert-informed, and patient-centered reporting framework. Although existing guidelines may recommend elements that are included in our framework, these recommendations are not always systematically implemented or reported. This consensus project may address the gap between guideline recommendations and real-world implementation.

This consensus project and its outcomes should still be considered in the context of certain limitations. Although we included an international cohort of experts, our sampling did not encompass all regions of the world, notably South America, and most of Asia and Africa were not represented. This geographic gap may limit the generalizability of our findings. To address this shortcoming, we plan to include these regions in the upcoming implementation phase. Our patient cohort consisted of 10 individuals, and while their diversity was ensured by use of a maximum variation sampling strategy, this sample size may not encompass the full spectrum of factors meaningful to patients. Because of the paucity of evidence for some topics, evidence-based statements were primarily based on a moderate-to-low LoE. A substantial challenge lies in the large proposed number of reporting elements and their integration into the clinical workflow. Questions arise regarding who will input data and at which time points, as well as what are the resources required for this reporting. Specifically, capturing long-term oncologic and quality of life outcomes is difficult to operationalize, even at the 1-year time point, given loss to follow-up and resource constraints. It is also crucial to acknowledge that some reporting elements included in the framework lacked evidence, which may hinder broad acceptance and implementation.

The next phase of the CoReAL project will solicit stakeholder feedback and address perceived challenges to implementation to improve the feasibility and utility of the reporting framework in clinical practice. Currently, the framework is undergoing evaluation by the American Society of Colon and Rectal Surgeons’ membership and other collaborating societies to gauge agreement with the reporting elements and potential adoption in the clinical setting. The research team is working on a large-scale implementation study by conducting interviews with health care workers from all over the world. The goal of these interviews is to identify the key obstacles to adoption, understand their perspectives, and gather insights on how to overcome these challenges. By addressing these barriers systematically, we aim to optimize the implementation process and ensure that the CoReAL framework is practical and valuable in everyday clinical practice. Further work is needed to evaluate the utility of the reporting framework using real-world clinical data sets, as well as the feasibility of data collection through electronic health records.

## CONCLUSIONS

The CoReAL project’s international collaborative consensus reporting framework represents an important advancement toward the standardization of reporting colorectal AL. By building on the highest LoE and incorporating diverse expert and patient perspectives, this framework may help to enhance the quality of reporting of AL, destigmatize leaks, and move our field toward a patient-centered approach that can lead to improved patient outcomes and future research.

## ACKNOWLEDGMENTS

Gregor Franssen was involved as a professional librarian from Maastricht University. The authors extend their gratitude to all the stakeholders who participated in the in-person consensus meeting organized by the American Society of Colon and Rectal Surgeons Research Foundation. They also wish to express appreciation once more to the CoReAL patient participants for their valuable input.

CoReAL collaborative: Michel Adamina, Alberto Arezzo, Mahdi Al-Taher, Tan Arulampalam, Saba Balvardi, Paul Barach, Himani Bhatt, Marta Botti, Stephanie O. Breukink, David A. Clark, Freek Daams, Jennifer S. Davids, Anse De Sadeleer, Abe Fingerhut, Zoe Garoufalia, Anke H.C. Gielen, Mukesh G. Harisinghani, Roel Hompes, Neil H. Hyman, Mehraneh D. Jafari, John T. Jenkins, Audrey C.H.M. Jongen, Deborah S. Keller, Samuel H. Lai, Jérémie H. Lefevre, Bibi Martens, Justin A. Maykel, Jeongyoon Moon, Nariaki Okomoto, Ian M. Paquette, Gianluca Pellino, Sherief F. Shawki, Benjamin D. Shogan, Chelliah Selvasekar, Simon Siu-Man Ng, Jasper Stijns, Patricia Tejedor, William Tzu-Liang Chen, Yu-Ting T. van Loon, Christiaan van Der Leij, Steven D. Wexner, Elizabeth Wick, and Marina Yiasemidou.

## Supplementary Material


