# Supplementary material for: International Consensus on Reporting Anastomotic Leaks After Colorectal Cancer Surgery: The CoReAL Reporting Framework
Source: Dis Colon Rectum. 2025 May 7;68(8):941–50. doi: 10.1097/DCR.0000000000003790 (PMC12269641; doi:10.1097/DCR.0000000000003790)
Supplement: Supplementary file 2 [file dcr-68-941-s002.pdf]

Appendix 1. Working groups and topic allocations

Topics	Experts		Surgical research collaborators
**Preoperative**	Nader Francis*	Jasper Stijns	Anse De Sadeleer
	Deborah Keller	Benjamin Shogan	Marta Botti
	Neil Hyman	Chelliah Selvasekar	
	Patricia Tejedor	Ian Paquette	
**Intraoperative**	Patricia Sylla*	Freek Daams	Danique Heuvelings
	Abe Fingerhut	Michel Adamina	Saba Balvardi
	Mahdi Al-Taher	Elizabeth Wick	Samuel Lai
	Simon NG Siu Man	Mehraneh Dorna Jafari	Zoe Garoufalia
	Sherief Shawki	Marina Yiasemidou	
**Postoperative short-term**	Marylise Boutros*	William Tzu-Liang Chen	Audrey Jongen
	Tina van Loon	Jeremie Lefevre	Nariaki Okamoto
	Jennifer Davids	David Clark	Himani Bhatt
	Ian Jenkins		Gianluca Pellino
**Postoperative long-term**	Nicole Bouvy*	Tan Arulampalam	Anke Gielen
	Stephanie Breukink	Roel Hompes	Jenny Moon
	Justin Maykel	Steven Wexner	
	Alberto Arrezzo		

*Team leads
