# Supplementary material for: International Consensus on Reporting Anastomotic Leaks After Colorectal Cancer Surgery: The CoReAL Reporting Framework
Source: Dis Colon Rectum. 2025 May 7;68(8):941–50. doi: 10.1097/DCR.0000000000003790 (PMC12269641; doi:10.1097/DCR.0000000000003790)
Supplement: Supplementary file 3 [file dcr-68-941-s003.pdf]

Supplementary CoReAL

Table S1. All research questions formulated by the coordinating team that were assessed during the literature search.

**1. Preoperative topics**
- What patient characteristics are preoperative risk factors for AL in colorectal cancer surgery?
- What is the effectiveness of prehabilitation versus no prehabilitation prior to elective colorectal cancer surgery on the percentage of AL?
- What is the effectiveness of mechanical bowel preparation versus no preparation prior to elective colorectal cancer surgery on the percentage of AL?
- What is the effectiveness of prophylactic oral antibiotics versus no ABX prior to colorectal cancer surgery on the percentage of AL?
- What is the effectiveness of anemia correction versus no correction prior to colorectal cancer surgery on the percentage of AL?
- What is the effectiveness of sarcopenia assessment versus no assessment prior to colorectal cancer surgery on the percentage of AL?
**2. Intraoperative topics**
- What is the comparative effectiveness of alternative operative technique (operations & anastomosis techniques) on the percentage of AL?
- What is the effectiveness of perfusion assessment versus no perfusion assessment during colorectal cancer surgery on the postoperative percentage of AL?
- What is the effectiveness of prophylactic diversion versus no prophylactic diversion during colorectal cancer surgery on the postoperative percentage of AL?
- What is the effectiveness of performing an integrity test versus no test during colorectal cancer surgery on the postoperative percentage of AL?
- What is the effectiveness of anastomotic reinforcement versus no reinforcement during colorectal cancer surgery on the postoperative percentage of AL?
- What is the effectiveness of placing a prophylactic drain versus no drain in the abdominal or pelvic cavity after colorectal cancer surgery on the percentage of AL?
- What is the effectiveness of an intraoperative ERAS protocol versus no protocol during colorectal cancer surgery on the percentage of AL?

- What is the effectiveness of transanal decompression tube versus no tube during low anterior colorectal cancer surgery on the percentage of AL?
- What is the effectiveness of using a validated intra-operative AL risk scoring system versus no scoring system during colorectal cancer surgery on the percentage of AL?
- What is the effectiveness of bypass devices versus no during colorectal cancer surgery on the postoperative percentage of AL?
- What is the influence of human factors during colorectal cancer surgery on the postoperative percentage of AL?

3. Postoperative short-term topics

- What is the diagnostic accuracy of validated clinical predictions scores for early identification of AL after colorectal cancer surgery?
- What is the diagnostic accuracy of biochemical markers on the percentage early detected ALs after colorectal surgery?
- What is the positive predictive value of postoperative imaging on the percentage of AL diagnoses after colorectal cancer surgery?
- What is the positive predictive value of endoscopic examination on the percentage of AL diagnoses after colorectal cancer surgery?
- What is the effectiveness of a low fiber diet versus regular diet postoperatively on the percentage of AL after colorectal cancer surgery?
- What is the effectiveness of postoperative prophylactic ABX versus no ABX on the percentage of AL after colorectal cancer surgery?
- What is the effectiveness of NSAID use versus no NSAID use on the percentage of AL after colorectal cancer surgery?
- Does the use of laxatives (e.g. movicolon or magnesiumhydroxide) versus no use of laxatives impact the percentage of AL?
- What is the effectiveness of an ERAS protocol versus no ERAS on the percentage early detected AL after colorectal cancer surgery? Does early discharge impact severity of AL after colorectal cancer surgery?
- What is the safety/feasibility of a minimal invasive approach versus open reintervention for AL after colorectal cancer surgery?
- What other approaches/techniques are present in the literature regarding reintervention for AL after colorectal cancer surgery?

4. **Postoperative long term topics**
- What is the oncologic impact of AL versus no AL following colorectal cancer surgery?
- What is the impact of AL versus no AL on quality of life after colorectal cancer surgery?
- What is the impact of AL versus no AL on the frequency of additional interventions for sequelae after colorectal cancer surgery?
- What is the impact of AL versus no AL on healthcare costs after colorectal cancer surgery?
- Are early and late leaks different identities?
- How should the impact of AL be measured after colorectal cancer surgery?
- When should we measure the impact of AL after colorectal cancer surgery?

Table S2. Search strategy

Database	Search Syntax
**Pubmed**	(((("Colorectal Neoplasms"[MeSH] OR (("Neoplasms"[MeSH] OR carcinoma*[tiab] OR adenocarcinoma*[tiab] OR neoplas*[tiab] OR tumour*[tiab] OR tumor*[tiab] OR oncolog*[tiab] OR malignan*[tiab] OR cancer*[tiab]) AND (colorectal*[tiab] OR colon[tiab] OR colonic[tiab] OR rectal[tiab] OR rectum[tiab] OR sigmoid*[tiab]))) AND ("Colectomy"[MeSH] OR "Colorectal Surgery"[MeSH] OR "Rectum/surgery"[MeSH] OR "Colon/surgery"[MeSH] OR ((large bowel[tiab] OR colorectal*[tiab] OR colon[tiab] OR rectum[tiab] OR rectal[tiab] OR ileocaecal[tiab] OR caecum[tiab] OR low anterior[tiab]) AND (resection*[tiab] OR surg*[tiab] OR anastomo*[tiab] OR "Anastomosis, Surgical"[MeSH])) OR (colectom*[tiab] OR hemicolectom*[tiab] OR "total mesorectal excision*"[tiab] OR proctocolectom*[tiab] OR "abdominal perineal resection*"[tiab]))) AND ("Anastomotic Leak"[MeSH] OR (anastomo*[tiab] AND ("adverse effects"[Subheading] OR "complications"[Subheading] OR leak*[tiab] OR complication*[tiab] OR defect*[tiab] OR separation*[tiab] OR dehiscence*[tiab] OR breakdown*[tiab] OR abscess*[tiab)))) AND (((systematic review[pt] OR (((systematic review[ti] OR systematic literature review[ti] OR systematic scoping review[ti] OR systematic narrative review[ti] OR systematic qualitative review[ti] OR systematic evidence

	review[ti] OR systematic quantitative review[ti] OR systematic meta-review[ti] OR systematic critical review[ti] OR systematic mixed studies review[ti] OR systematic mapping review[ti] OR systematic cochrane review[ti] OR systematic search and review[ti] OR systematic integrative review[ti]) NOT comment[pt] NOT (protocol[ti] OR protocols[ti])) NOT MEDLINE [subset]) OR (Cochrane Database Syst Rev[ta] AND review[pt])) OR ("Meta-Analysis"[pt] OR meta analysis[ti])) OR ("Randomized Controlled Trial"[pt] OR "Controlled Clinical Trial"[pt] OR ((random*[tiab] AND (controlled[tiab] OR control[tiab] OR placebo[tiab] OR versus[tiab] OR vs[tiab] OR group[tiab] OR groups[tiab] OR comparison[tiab] OR compared[tiab] OR crossover[tiab] OR cross-over[tiab]) AND (trial[tiab] OR study[tiab])) OR ((single[tiab] OR double[tiab] OR triple[tiab]) AND (masked[tiab] OR blind*[tiab])))))) NOT ((("Animals"[MeSH]) OR "Models, Animal"[MeSH] NOT "Humans"[MeSH]) NOT (letter[pt] OR comment[pt] OR editorial[pt]))
**Embase**	(exp colorectal tumor/ or exp colorectal cancer/ or ((neoplasm/ or (carcinoma* or adenocarcinoma* or neoplas* or tumour* or tumor* or oncolog* or malignan* or cancer*).ti,ab,kw.) adj3 (colorectal* or colon or colonic or rectal or rectum or sigmoid*).ti,ab,kw.)) and (exp colorectal surgery/ or exp rectum surgery/ or exp colon surgery/ or ileoanal anastomosis/ or ileorectal anastomosis/ or ((large bowel or colorectal* or colon or rectum or rectal or ileocaecal or caecum or low anterior).ti,ab,kw. adj3 (resection* or surg* or anastomo*).ti,ab,kw. or (colectom* or hemicolectom* or "total mesorectal excision*" or proctocolectom* or "abdominal perineal resection*").ti,ab,kw.)) and (postoperative complication/su or exp anastomosis leakage/ or anastomosis/co or (anastomo* adj3 (leak* or complication*)).ti,ab,kw.) and ((("systematic review"/ or (systematic review.ti. or systematic literature review.ti. or systematic scoping review.ti. or systematic narrative review.ti. or systematic qualitative review.ti. or systematic evidence review.ti. or systematic quantitative review.ti. or systematic meta-review.ti. or systematic critical review.ti. or systematic mixed studies review.ti. or systematic mapping review.ti. or systematic cochrane review.ti. or "systematic search and review".ti. or systematic integrative review.ti.)) or (meta analysis/ or meta

	analysis.ti.) or (randomized controlled trial/ or ((random*.ti,ab. and (controlled.ti,ab. or control.ti,ab. or placebo.ti,ab. or versus.ti,ab. or vs.ti,ab. or group.ti,ab. or groups.ti,ab. or comparison.ti,ab. or compared.ti,ab. or crossover.ti,ab. or cross-over.ti,ab.) and (trial.ti,ab. or study.ti,ab.)) or ((single.ti,ab. or double.ti,ab. or triple.ti,ab.) and (masked.ti,ab. or blind*.ti,ab.))))) NOT ((exp animal/ or nonhuman/) NOT exp human/) NOT (letter or editorial).pt.)
**Cochrane**	("Colorectal Neoplasms"[MeSH] OR (("Neoplasms"[MeSH] OR carcinoma*:ti,ab,kw OR adenocarcinoma*:ti,ab,kw OR neoplas*:ti,ab,kw OR tumour*:ti,ab,kw OR tumor*:ti,ab,kw OR oncolog*:ti,ab,kw OR malignan*:ti,ab,kw OR cancer*:ti,ab,kw) AND (colorectal*:ti,ab,kw OR colon:ti,ab,kw OR colonic:ti,ab,kw OR rectal:ti,ab,kw OR rectum:ti,ab,kw OR sigmoid*:ti,ab,kw))) AND ("Colectomy"[MeSH] OR "Colorectal Surgery"[MeSH] OR "Rectum/surgery"[MeSH] OR "Colon/surgery"[MeSH] OR ((large bowel:ti,ab,kw OR colorectal*:ti,ab,kw OR colon:ti,ab,kw OR rectum:ti,ab,kw OR rectal:ti,ab,kw OR ileocaecal:ti,ab,kw OR caecum:ti,ab,kw OR low anterior:ti,ab,kw) AND (resection*:ti,ab,kw OR surg*:ti,ab,kw OR anastomo*:ti,ab,kw OR "Anastomosis, Surgical"[MeSH])) OR (colectom*:ti,ab,kw OR hemicolectom*:ti,ab,kw OR "total mesorectal excision*":ti,ab,kw OR proctocolectom*:ti,ab,kw OR "abdominal perineal resection*":ti,ab,kw)) AND ("Anastomotic Leak"[MeSH] OR (anastomo*:ti,ab,kw AND ("adverse effects"[Subheading] OR "complications"[Subheading] OR leak*:ti,ab,kw OR complication*:ti,ab,kw OR defect*:ti,ab,kw OR separation*:ti,ab,kw OR dehiscence*:ti,ab,kw OR breakdown*:ti,ab,kw OR abscess*:ti,ab,kw))

S3. Expert commentary on the statements

- Statement 7: Although the statement on sarcopenia based on the available evidence, experts believe sarcopenia may be associated with AL and future research needs to be performed to investigate the impact of sarcopenia and frailty to AL outcomes.
- Statement 9: Splenic flexure mobilization represents a way to create a tension free anastomosis. The experts asked themselves ‘What is tension free?’ and concluded this cannot be measured objectively. They therefore decided to formulate the statement

regarding flexure mobilization, with the expert note that if there is no tension free anastomosis, the risk of AL increases and flexure mobilization is a way to reduce this risk, but, as stated here, this is not necessary to perform routinely.

- Statement 11: As conversion reflects intraoperative difficulty, it is not the conversion itself that increases AL rates, but reflects the fact that the operation was difficult, which is a risk factor for AL development.
- Statement 15: The ‘severity’ of leaks is something difficult to measure, but the experts decided to use this phrasing as it’s more about the consequences of leaks instead of the rates.
- Statement 27: The experts state that transanal or endoscopic management is possible when appropriate expertise is available. Besides, some experts stated that this should never be performed alone but always in combination with a lavage, although this opinion was not supported by everyone, nor was this specified in the evidence.
