# Supplementary material for: International Consensus on Reporting Anastomotic Leaks After Colorectal Cancer Surgery: The CoReAL Reporting Framework
Source: Dis Colon Rectum. 2025 May 7;68(8):941–50. doi: 10.1097/DCR.0000000000003790 (PMC12269641; doi:10.1097/DCR.0000000000003790)
Supplement: Supplementary file 4 [file dcr-68-941-s004.pdf]

Appendix 2. Detailed reporting framework

Preoperative reporting elements

**Modifiable risk factors**	**Other risk factors**	**Mechanical bowel preparation**
☐ Alcohol: ……/ week<br>☐ Active smoker: …… cigarettes / day<br>☐ Serum albumin level < 30 days before the surgery: ……	☐ Drop down menu<br>○ BMI<br>○ Gender: male / female<br>○ ASA score<br>○ Diabetes: yes (Hba1c:….) / no<br>○ Cardiovascular disease: yes / no<br>○ Chronic renal failure or insufficiency: yes / no<br>○ Chronic steroid use: yes / no<br>○ Clinical T-stage: T1 or T2 (not advanced) / T3 or T4 (advanced)<br>○ Tumor size: > 5cm / < 5 cm<br>○ Tumor location: right / transverse / left / rectal<br>○ Complicated tumor: yes (perforation / obstruction) / no<br>○ Neoadjuvant therapy: preop chemotherapy / radiotherapy / long course chemotherapy / short course chemotherapy / immunotherapy	☐ Yes<br>☐ No
**Was the patient referred to a stoma therapist preoperatively?**	**Was the potential need of a postoperative/permanent stoma discussed?**	**Preoperative oral antibiotics**
☐ Yes<br>☐ No	☐ Yes<br>☐ No	☐ Yes, Specify: ……<br>☐ No

Intraoperative reporting elements

**Conversion MIS to open**	**Number of staple loads for rectal transection**	**Perfusion assessment of conduit with fluorescence angiography**	**Anastomotic integrity testing**	**Location of inferior mesenteric artery ligation**
☐ Yes<br>☐ No<br>☐ N/A = open	☐ N/A = handsewn<br>☐ 0<br>☐ 1<br>☐ 2<br>☐ >2	☐ Yes<br>☐ No	☐ Yes<br>Consequence: …<br>☐ No	☐ Distal to the left colic artery<br>☐ Proximal to the left colic artery
**Pitfalls**	**Diverting stoma creation**	**Splenic flexure mobilization**	**Intraoperative difficulty**	
Critical events<br>☐ Pelvic stapler failure<br>☐ Other device failures<br>☐ Unplanned multivisceral* resection or repair	☐ No<br>☐ Yes<br>☐ unplanned<br>☐ planned<br>○ ileostomy<br>○ colostomy	☐ Yes<br>☐ No	☐ Distance of the anastomosis (cm) from AV<br>☐ Acute blood loss requiring intraop blood transfusion<br>☐ Redo pelvic surgery	

Postoperative early (surgery – 90 days) reporting elements

<table>
<tr><td colspan="2">Before discharge (index admission)
POD of AL diagnosis: …… days
LOS: …… days
ICU LOS: …… days</td><td colspan="2">Mortality:
☐ Yes: POD ……
☐ No</td></tr>
<tr><th>Serial CRP measurement</th><th>Mode of diagnosis</th><th>Re-interventions until discharge</th><th>Stoma creation</th></tr>
<tr><td>☐ Yes
☐ No</td><td>☐ No
☐ Yes
→ Drop down menu:
☐ CT scan - POD: ……
Contrast: PO / IV / rectal / NA
☐ Endoscopy - POD: ……
☐ Contrast enema - POD: ……
☐ Surgical - POD: ……
○ EUA
○ Transabdominal: minimal invasive / open</td><td>☐ None
☐ Yes
→ Drop down menu:
☐ Antibiotics
☐ Radiological drainage - POD: ……
○ Trans-abdominal
○ Trans-gluteal
☐ Transanal/endoscopic repair - POD: ……
○ Endosponge
○ Repair
○ Clipping
○ Drainage
○ Dilatation</td><td>☐ NA
☐ Yes
○ Ileostomy
○ Loop
○ End
○ Colostomy
○ Loop
○ End</td></tr>
</table>

		☐ Abdominal: minimal invasive / open - POD: ……<br>○ Takedown<br>○ Repair<br>○ Redo<br>○ Drainage<br>○ Tissue flap<br>○ Stoma creation<br>☐ Other: ……		
**After discharge – 30 days AND 90 days**<br>☐ Not applicable<br>POD of AL diagnosis: …… days		Mortality:<br>☐ Yes: POD ……<br>☐ No		
**Readmission**	**Diagnostic modality**	**Re-interventions after initial discharge**	**Stoma**	**Anastomotic complication**
☐ Yes<br>LOS: …… days<br>ICU LOS: …… days<br>☐ No	☐ N/A<br>☐ CT scan - POD: ……<br>Contrast: PO / IV / rectal / NA<br>☐ Endoscopy - POD: ……<br>☐ Contrast enema - POD: ……<br>☐ Surgical - POD: ……<br>○ EUA	☐ None<br>☐ Antibiotics<br>☐ Radiological drainage - POD: ……<br>○ Trans-abdominal<br>○ Trans-gluteal<br>☐ Transanal/endoscopic repair - POD: ……<br>○ Endosponge	**Creation**<br>☐ NA<br>☐ Yes<br>○ Ileostomy<br>○ Loop<br>○ End<br>○ Colostomy<br>○ Loop	☐ Resolved/None<br>☐ Leak<br>○ Defect, dehiscence or sinus<br>○ Stricture or stenosis

	○ Transabdominal: minimal invasive / open	○ Repair<br>○ Clipping<br>○ Drainage<br>○ Dilatation<br>☐ Abdominal: minimal invasive / open - POD: ……<br>○ Takedown<br>○ Repair<br>○ Redo<br>○ Drainage<br>○ Tissue flap<br>○ Stoma creation<br>☐ Other: ……	○ End<br><br>**Stoma closure**<br>☐ N/A<br>☐ Yes: POD ……<br>☐ Reversal planned<br>☐ No:<br>○ Ongoing leak<br>○ Medically ineligible<br>○ Ongoing cancer tx<br>○ Patient declined<br>○ Other: ……	○ Fistula<br>○ Osteomyelitis

Postoperative late (90+ days) reporting elements

**Re-interventions after 90 days**<br>*Until 1 year*	**Anastomotic status**<br>*Until 1 year*	**Stoma**<br>*Until 1 year*	**Anastomotic complications**<br>*Until 1 year*
☐ No<br>☐ Yes<br>→ *Drop down menu:*<br>☐ Antibiotics<br>☐ Radiological drainage - POD: ……<br>○ Trans-abdominal<br>○ Trans-gluteal<br>☐ Transanal/endoscopic repair - POD: ……<br>○ Endosponge<br>○ Repair<br>○ Clipping<br>○ Drainage<br>○ Dilatation<br>☐ Abdominal: minimal invasive / open - POD: ……<br>○ Takedown<br>○ Repair<br>○ Redo<br>○ Drainage	☐ Primary<br>☐ Secondary<br><br>☐ Healed<br>☐ Not healed	☐ No stoma<br>☐ Reversal planned<br>☐ Stoma<br>→ *Drop down menu:*<br>○ Ileostomy<br>○ Loop<br>○ End<br>○ Colostomy<br>○ Loop<br>○ End<br>Reason:<br>○ Ongoing leak<br>○ Medically ineligible<br>○ Ongoing cancer tx<br>○ Patient declined<br>○ Other: ……<br>○ Reversal planned	☐ Resolved/None<br>☐ Leak<br>○ Defect, dehiscence or sinus<br>○ Stricture or stenosis<br>○ Fistula<br>○ Osteomyelitis

○ Tissue flap<br>○ Stoma creation<br>☐ Other: ……			
**Oncological outcomes**<br>*1 year – 2 years – 5 years*	**Functional outcomes**<br>*1 year – 2 years – 5 years*	**Quality of Life**<br>*1 year – 2 years – 5 years*	**Mortality**<br>*1 year – 2 years – 5 years*
Local recurrence<br>☐ Yes<br>☐ No<br>Distant recurrence<br>☐ Yes<br>☐ No	☐ LARS - LARS score ……<br>☐ Incontinence - Wexner FI score ……	☐ EQ-5D score	☐ No<br>☐ Yes: POD ……
